# Consensus guidelines for the definition, detection and interpretation of immunogenic cell death

**DOI:** 10.1136/jitc-2019-000337

**Published:** 2020-03-09

**Authors:** Lorenzo Galluzzi, Ilio Vitale, Sarah Warren, Sandy Adjemian, Patrizia Agostinis, Aitziber Buqué Martinez, Timothy A Chan, George Coukos, Sandra Demaria, Eric Deutsch, Dobrin Draganov, Richard L Edelson, Silvia C Formenti, Jitka Fucikova, Lucia Gabriele, Udo S Gaipl, Sofia R Gameiro, Abhishek D Garg, Encouse Golden, Jian Han, Kevin J Harrington, Akseli Hemminki, James W Hodge, Dewan Md Sakib Hossain, Tim Illidge, Michael Karin, Howard L Kaufman, Oliver Kepp, Guido Kroemer, Juan Jose Lasarte, Sherene Loi, Michael T Lotze, Gwenola Manic, Taha Merghoub, Alan A Melcher, Karen L Mossman, Felipe Prosper, Øystein Rekdal, Maria Rescigno, Chiara Riganti, Antonella Sistigu, Mark J Smyth, Radek Spisek, John Stagg, Bryan E Strauss, Daolin Tang, Kazuki Tatsuno, Stefaan W van Gool, Peter Vandenabeele, Takahiro Yamazaki, Dmitriy Zamarin, Laurence Zitvogel, Alessandra Cesano, Francesco M Marincola

**Affiliations:** 1 Department of Radiation Oncology, Weill Cornell Medical College, New York City, New York, USA; 2 Sandra and Edward Meyer Cancer Center, New York City, New York, USA; 3 Caryl and Israel Englander Institute for Precision Medicine, New York City, New York, USA; 4 Department of Dermatology, Yale School of Medicine, New Haven, Connecticut, USA; 5 Université de Paris, Paris, France; 6 IIGM - Italian Institute for Genomic Medicine, c/o IRCSS, Candiolo, Torino, Italy; 7 Candiolo Cancer Institute, FPO - IRCCS, Candiolo, Italy; 8 NanoString Technologies, Seattle, Washington, USA; 9 VIB Center for Inflammation Research (IRC), Ghent, Belgium; 10 Department of Biomedical Molecular Biology (DBMB), Ghent University, Ghent, Belgium; 11 Cell Death Research & Therapy (CDRT) Laboratory, Department of Cellular and Molecular Medicine, KU Leuven, Leuven, Belgium; 12 VIB-KU Leuven Center for Cancer Biology, KU Leuevn, Leuven, Belgium; 13 Human Oncology and Pathogenesis Program, Memorial Sloan Kettering Cancer Center, New York City, New York, USA; 14 Immunogenomics and Precision Oncology Platform, Memorial Sloan Kettering Cancer Center, New York City, New York, USA; 15 Ludwig Institute for Cancer Research and Department of Oncology, University of Lausanne, Lausanne, Switzerland; 16 Department of Pathology and Laboratory Medicine, Weill Cornell Medical College, New York City, New York, USA; 17 Department of Radiation Oncology, Gustave Roussy Cancer Campus, Villejuif, France; 18 INSERM "Molecular Radiotherapy and therapeutic innovation", U1030 Molecular Radiotherapy, Gustave Roussy Cancer Campus, Villejuif, France; 19 SIRIC SOCRATES, DHU Torino, Faculté de Medecine, Université Paris-Saclay, Kremlin-Bicêtre, France; 20 Calidi Biotherapeutics, San Diego, California, USA; 21 Comprehensive Cancer Center, Yale University School of Medicine, New Haven, Connecticut, USA; 22 Department of Immunology, Charles University, 2nd Faculty of Medicine and University Hospital Motol, Prague, Czech Republic; 23 Sotio, Prague, Czech Republic; 24 Department of Oncology and Molecular Medicine, Istituto Superiore di Sanità, Rome, Italy; 25 Universitätsklinikum Erlangen, Erlangen, Germany; 26 Laboratory of Tumor Immunology and Biology, National Cancer Institute/Center for Cancer Research, National Institutes of Health, Bethesda, Maryland, USA; 27 iRepertoire, Inc, Huntsville, Alabama, USA; 28 Division of Radiotherapy and Imaging, The Institute of Cancer Research, London, UK; 29 The Royal Marsden Hospital/Institute of Cancer Research National Institute for Health Biomedical Research Centre, London, UK; 30 Cancer Gene Therapy Group, Translational Immunology Research Program, University of Helsinki, Helsinki, Finland; 31 Comprehensive Cancer Center, Helsinki University Hospital, Helsinki, Finland; 32 Merck & Co. Inc, Kenilworth, New Jersey, USA; 33 University of Manchester, NIHR Manchester Biomedical Research Centre, Christie Hospital, Manchester, UK; 34 Department of Pharmacology and Pathology, University of California at San Diego (UCSD) School of Medicine, La Jolla, California, USA; 35 Division of Surgical Oncology, Massachusetts General Hospital, Boston, Massachusetts, USA; 36 Replimune, Inc, Woburn, Massachusetts, USA; 37 Metabolomics and Cell Biology Platforms, Gustave Roussy Comprehensive Cancer Institute, Villejuif, France; 38 Equipe 11 labellisée Ligue contre le Cancer, Centre de Recherche des Cordeliers, Paris, France; 39 Gustave Roussy Comprehensive Cancer Institute, Villejuif, France; 40 INSERM, U1138, Paris, France; 41 Sorbonne Université, Paris, France; 42 Pôle de Biologie, Hôpital Européen Georges Pompidou, AP-HP, Paris, France; 43 Karolinska Institute, Department of Women’s and Children’s Health, Karolinska University Hospital, Stockholm, Sweden; 44 Suzhou Institute for Systems Medicine, Chinese Academy of Medical Sciences, Suzhou, China; 45 Program of Immunology and Immunotherapy, Centro de Investigación Médica Aplicada (CIMA), University of Navarra, Pamplona, Spain; 46 Division of Research and Clinical Medicine, Peter MacCallum Cancer Centre, Melbourne, Victoria, Australia; 47 Department of Oncology, University of Melbourne, Parkville, Victoria, Australia; 48 Department of Surgery, University of Pittsburgh School of Medicine, Pittsburgh, Pennsylvania, USA; 49 Department of Immunology, University of Pittsburgh School of Medicine, Pittsburgh, Pennsylvania, USA; 50 Department of Bioengineering, University of Pittsburgh School of Medicine, Pittsburgh, Pennsylvania, USA; 51 Ludwig Collaborative and Swim Across America Laboratory, MSKCC, New York City, New York, USA; 52 Weill Cornell Medical College, New York City, New York, USA; 53 Parker Institute for Cancer Immunotherapy, MSKCC, New York City, New York, USA; 54 The Institute of Cancer Research, London, UK; 55 McMaster University, Hamilton, Ontario, Canada; 56 Hematology and Cell Therapy, Clinica Universidad de Navarra, Pamplona, Spain; 57 Lytix Biopharma, Oslo, Norway; 58 Department of Medical Biology, University of Tromsø, Tromsø, Norway; 59 Humanitas Clinical and Research Center – IRCCS, Rozzano, Italy; 60 Humanitas University, Department of Biomedical Sciences, Pieve Emanuele, Milan, Italy; 61 Department of Oncology, University of Torino, Torino, Italy; 62 Interdepartmental Research Center of Molecular Biotechnology, University of Torino, Torino, Italy; 63 UOSD Immunology and Immunotherapy Unit, IRCCS Regina Elena National Cancer Institute, Rome, Italy; 64 Istituto di Patologia Generale, Università Cattolica del Sacro Cuore, Rome, Italy; 65 Immunology in Cancer and Infection Laboratory, QIMR Berghofer Medical Research Institute, Herston, Queensland, Australia; 66 Centre de Recherche du Centre Hospitalier de l’Université de Montréal (CRCHUM), Montréal, Quebec City, Canada; 67 Institut du Cancer de Montréal, Montréal, Quebec City, Canada; 68 Faculté de Pharmacie de l’Université de Montréal, Montréal, Quebec City, Canada; 69 Centro de Investigação Translacional em Oncologia/LIM24, Instituto do Câncer do Estado de São Paulo, Faculdade de Medicina, Universidade de São Paulo, São Paulo, Brasil; 70 Department of Surgery, UT Southwestern Medical Center, Dallas, Texas, USA; 71 Immun-Onkologisches Zentrum Köln, Cologne, Germany; 72 Methusalem program, Ghent University, Ghent, Belgium; 73 Department of Medicine, Weill Cornell Medical College, New York City, New York, USA; 74 Department of Medicine, Memorial Sloan Kettering Cancer Center, New York City, New York, USA; 75 Equipe labellisée par la Ligue contre le cancer, Gustave Roussy, Villejuif, France; 76 Faculty of Medicine, University of Paris Sud/Paris Saclay, Le Kremlin-Bicêtre, France; 77 INSERM U1015, Villejuif, France; 78 Center of Clinical Investigations in Biotherapies of Cancer (CICBT) 1428, Villejuif, France; 79 ESSA Pharmaceuticals, South San Francisco, California, USA; 80 Refuge Biotechnologies, Menlo Park, California, USA

**Keywords:** oncology, immunology, molecular biology

## Abstract

Cells succumbing to stress via regulated cell death (RCD) can initiate an adaptive immune response associated with immunological memory, provided they display sufficient antigenicity and adjuvanticity. Moreover, multiple intracellular and microenvironmental features determine the propensity of RCD to drive adaptive immunity. Here, we provide an updated operational definition of immunogenic cell death (ICD), discuss the key factors that dictate the ability of dying cells to drive an adaptive immune response, summarize experimental assays that are currently available for the assessment of ICD in vitro and in vivo, and formulate guidelines for their interpretation.

## Introduction

Regulated cell death (RCD), a form of cellular demise that is governed by a genetically encoded molecular machinery,^1 2^ has long been considered as an immunologically silent or even tolerogenic event.^3^ At least in part, this widely accepted view originated from the highly tolerogenic nature of programmed cell death (PCD), the physiological variant of RCD that contributes to postembryonic development and adult tissue turnover.^1 4^ However, it has now become clear that, at least under specific circumstances, stress-induced RCD can drive an inflammatory response that may culminate with the activation of cytotoxic T lymphocyte (CTL)-driven adaptive immunity coupled with the establishment of long-term immunological memory. Such a functionally unique form of stress-driven RCD is now usually referred to as immunogenic cell death (ICD).^5^

Cellular stressors that are associated with ICD encompass (but are not limited to): (1) obligate intracellular pathogens including multiple bacterial and viral species^6–8^; (2) therapeutic oncolytic viruses^9–16^; (3) various molecules with oncolytic potential^17–19^; (4) conventional chemotherapeutics such as numerous anthracyclines (ie, doxorubicin, epirubicin, idarubicin and mitoxantrone), some (but importantly not all) DNA-damaging agents (ie, cyclophosphamide and oxaliplatin, but not cisplatin), poly-A-ribose polymerase (PARP) inhibitors, mitotic poisons (ie, docetaxel and patupilone) and proteasomal inhibitors (ie, bortezomib and carfilzomib)^20–25^; (5) epigenetic modifiers including DNA methyltransferase, histone deacetylase (HDAC) and bromodomain inhibitors^26–30^; (6) targeted anticancer agents such as the tyrosine kinase inhibitor crizotinib, the epidermal growth factor receptor (EGFR)-specific monoclonal antibody cetuximab, the cyclin-dependent kinase (CDK) inhibitor dinaciclib and the Bruton tyrosine kinase (BTK) inhibitor ibrutinib^31–33^; (7) other chemicals including the ubiquitin-specific peptidase inhibitor spautin-1, the antibiotic bleomycin, the protein phosphatase-2A inhibitor LB-100, the Chinese herbal medicine component shikonin and capsaicin^34–38^ and (8) numerous physical interventions, encompassing various forms of ionizing radiation, extracorporeal photochemotherapy, hypericin-based photodynamic therapy (PDT), near‐infrared photoimmunotherapy, high hydrostatic pressure, severe cytotoxic heat shock, nanopulse stimulation and electrohyperthermia.^39–49^ Importantly, dose and administration schedules have a major impact on the ability of many of these agents to initiate productive ICD.^50–52^

The aforementioned ICD inducers have been instrumental not only for identifying the molecular machinery that underlies the immunogenicity of some variants of RCD,^5^ but also for elucidating the pathophysiological and therapeutic implications of the process.^53^ Indeed, the ability of ICD to initiate adaptive immunity not only is critical for the optimal eradication of infectious pathogens,^54^ but also influences the cancer-immunity cycle by tipping the balance toward antitumor immunity.^55^ Consistent with this notion, both pathogens and progressing tumors harness strategies that enable immunoevasion by avoiding ICD induction.^5^ Moreover, accumulating clinical evidence demonstrates that numerous ICD inducers commonly employed in the management of cancer patients synergize with immunotherapy with immune checkpoint blockers (ICBs), as long as they do not compromise immunostimulatory signals or the activity of tumor-infiltrating lymphocytes.^56 57^

The morphological features displayed by dying cells and the molecular mechanisms that are mechanistically responsible for the cellular demise do not necessarily correlate with the immunogenicity of RCD.^58^ Thus, while specific instances of caspase 3 (CASP3)-dependent apoptosis and mixed lineage kinase domain-like pseudokinase (MLKL)-dependent necroptosis initiate adaptive immunity in certain experimental settings,^20 59 60^ RCD accompanied by CASP3 or MLKL activation is not necessarily immunogenic.^61–63^ Moreover, while the perception of RCD as immunogenic has been etiologically attributed to the emission of specific signals from dying cells (see *Definition of immunogenic cell death*), the presence of such signals is not necessarily predictive of the ability of dying cells to drive adaptive immunity in vivo.^34 64^ Altogether, these observations highlight the importance of defining standardized experimental settings that enable the assessment of ICD in the context of a robust conceptual framework for the interpretation of results. Here, we provide a general overview of the factors that underpin the immunogenicity or RCD, and attempt to provide such a framework by formulating guidelines for the definition, detection and interpretation of ICD.

### Definition of ICD

The Nomenclature Committee on Cell Death has recently defined ICD as ‘a form of RCD that is sufficient to activate an adaptive immune response in immunocompetent syngeneic hosts’,^1^ which properly reflects the two major components of ICD as a process, that is, the cellular component and the host component. Importantly, the latter does not refer to potential defects of the host that prevent the initiation of adaptive immunity (eg, HLA mismatch, systemic immunodeficiency), but to features intrinsic to dying cells that render them immunogenic only in specific hosts. Indeed, the ability of RCD to drive adaptive immunity depends on two major parameters, neither of which is ultimately intrinsic to dying cells: antigenicity and adjuvanticity.

Antigenicity is conferred by the expression and presentation of antigens that fail to induce clonal deletion in the context of central tolerance in a specific host, implying that the host contains naïve T cell clones that can recognize such antigens.^65 66^ Thus, healthy cells are limited in their ability to drive ICD, as their antigens are typically expressed by the thymic epithelium during T cell development. As an exception, some naïve T cell clones expressing self-reactive low-affinity T cell receptors (TCRs) escape thymic selection, implying that such antigens may support ICD in the context of peripheral tolerance breakdown (see *Sources of ICD antigenicity*). Conversely, infected cells, as well as malignant cells, display sufficient antigenicity to drive immune responses, as they express a panel of antigenic epitopes for which naïve T cell clones are generally available. These antigenic determinants include neoepitopes that are highly immunogenic as they are not covered by central tolerance as well as (non-mutated) epitopes that may be immunogenic due to gaps in central tolerance and/or incomplete peripheral tolerance.^67^ Adjuvanticity is provided by the spatiotemporally coordinated release or exposure of danger signals that are necessary for the recruitment and maturation of antigen-presenting cells (APCs), which are cumulatively referred to as damage-associated molecular patterns (DAMPs).^68 69^ Although most (if not all) cells contain DAMPs in levels that are sufficient to drive robust APC stimulation, the kinetics and intensity of their release are dictated by intracellular responses driven by the initiating stressor.^70–72^ This may explain why some cytotoxic agents can drive ICD while others are unable to, despite their similar RCD-inducing capability.^23 73^

Microenvironmental conditions also have a dramatic influence on the propensity of infected or neoplastic cells undergoing a potentially immunogenic variant of RCD to initiate adaptive immunity and/or be susceptible to CTL-dependent lysis, thus impacting both the priming and the effector phase of the immunological response. As an example, mouse cancer cells irradiated in vitro can be successfully employed to immunize immunocompetent syngeneic mice against a subsequent challenge with living cells of the same type, demonstrating the elicitation of immunological memory.^74^ Conversely, in therapeutic settings, the immunosuppressive microenvironment that characterizes a majority of tumors can considerably limit ICD-driven immunity.^75 76^ Thus, irradiating a neoplastic lesion established in immunocompetent, syngeneic mice generally fails to generate a CTL-dependent immune response of sufficient strength to eradicate a distant, non-irradiated lesion, unless additional immunostimulatory molecules are provided.^77–79^

**Figure 1:**
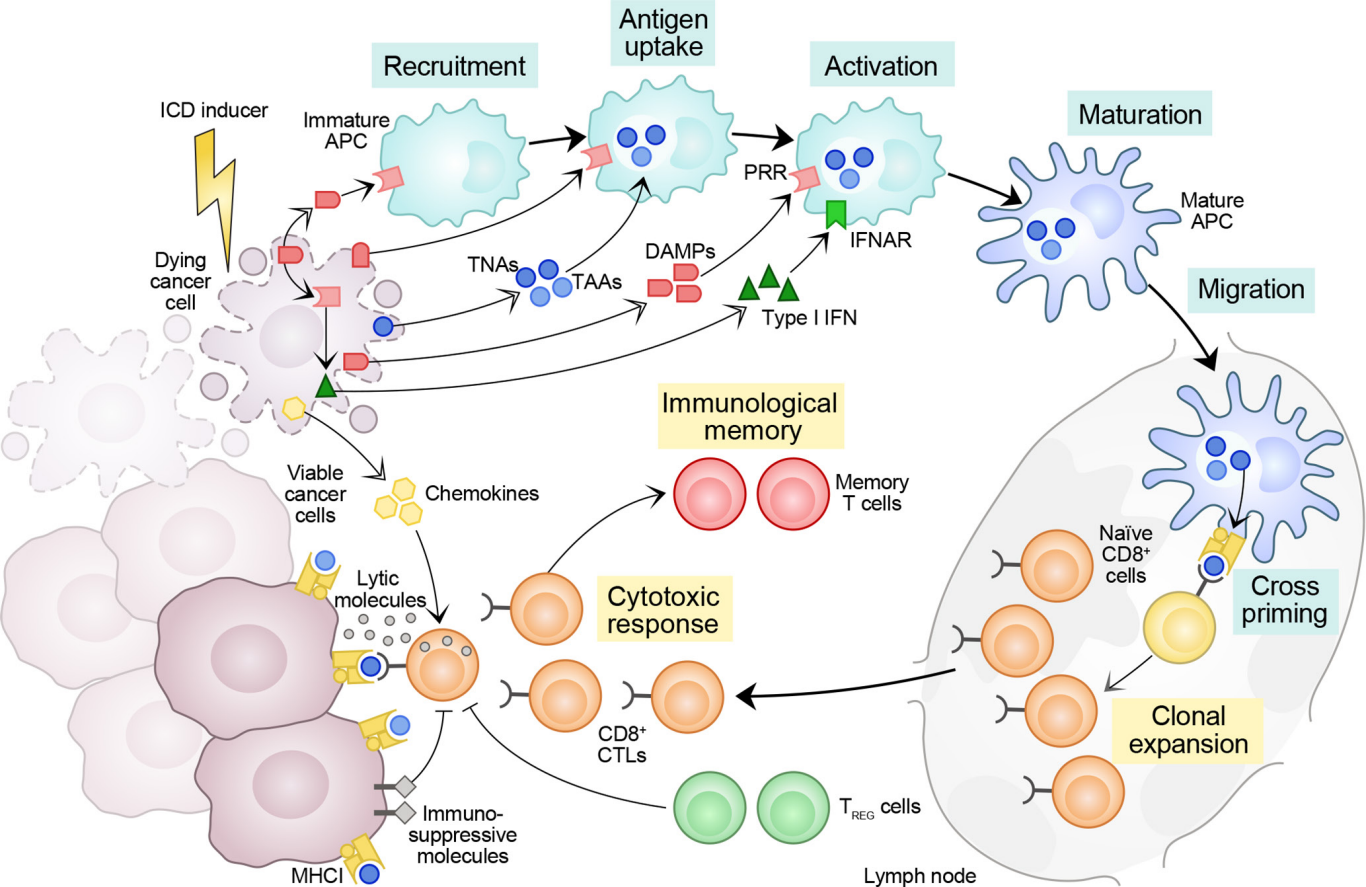
Major factors dictating the immunogenicity of cell death. Cells undergoing regulated cell death (RCD) in response to stress can prime an adaptive immune response specific for dead cell-associated antigens provided that (1) those antigens are not perfectly covered by central tolerance, and (2) dying cells emit a panel of immunostimulatory damage-associated molecular patterns (DAMPs) and cytokines that, when delivered according to a precise spatiotemporal pattern, support the recruitment, phagocytic activity and maturation of antigen-presenting cells (APCs), de facto enabling them to engulf antigenic material, migrate to lymph nodes and prime a cytotoxic T lymphocyte (CTL)-dependent immune response. As they express tumor neoantigens (TNAs, which are not covered by central tolerance) and/or tumor-associated antigens (TAAs, for which central tolerance is leaky), cancer cells can undergo bona fide immunogenic cell death (ICD) in response to select stimuli, including (but not limited to) some chemotherapeutic agents commonly employed in the clinic, as well as radiation therapy. However, the TME is generally characterized by an immunosuppressive profile that may prevent either the initiation or the execution of ICD-driven anticancer immunity. Thus, the ultimate ability of RCD to drive adaptive immunity does not depend only on the initiating stimulus and the dying cell, but also on features that are intrinsic to the host. IFNAR, interferon-alpha/beta receptor; PRR, pattern recognition receptor; T_REG_, regulatory T; TME, tumor microenvironment.

Taken together, these observations suggest that while the adjuvanticity of RCD depends on dying cells and the capability of the initiating stressor to elicit danger signaling, its immunogenicity ultimately depends on dying cells and the host, which also determines the propensity of dying cells to drive adaptive immunity at the microenvironmental level (figure 1).

### Sources of ICD antigenicity

Infection by pathogenic microbes is an obvious source of antigenic determinants, as microbial proteins are not covered by central tolerance and hence their epitopes are highly antigenic.^80 81^ Together with the ability of conserved microbial products cumulatively referred to as microbe-associated molecular patterns (MAMPs) to deliver potent immunostimulatory signals, this explains why RCD driven by intracellular pathogens is highly immunogenic.^82–84^ The same generally does not apply to healthy syngeneic cells, as thymic and peripheral tolerance result in the deletion or functional inactivation of self-reactive T cell clones from the mature host T cell repertoire,^85^ although some naïve T cell clones expressing self-reactive low-affinity TCRs can escape thymic selection and hence drive (especially in the context of lost peripheral tolerance) autoimmune reactions.^86^

There are at least two exceptions to this principle, which may underlie the ability of healthy cells to undergo ICD. First, the genome of normal cells contains a significant number of endogenous retroviruses, which are generally latent (ie, not transcribed) in physiological conditions.^87 88^ In response to some cellular stressors, however, endogenous retroviruses can become activated and/or retroviral genes can be expressed, resulting in the synthesis of potentially antigenic proteins.^89^ Second, antigenic determinants can be generated by enzymatic or non-enzymatic post-translational modifications (PTMs) that alter protein structure, encompassing (but perhaps not limited to) phosphorylation, acetylation, glycosylation, citrullination, nitration/nitrosylation, glycation, oxidation and ubiquitination.^90^ Moreover, the antigenic peptide repertoire can be boosted by alterations in the activation of reticular aminopeptidases such as endoplasmic reticulum aminopeptidase 1 (ERAP1) and ERAP2.^91^ Importantly, the signal transduction cascades that regulate enzymatic PTMs are sensitive to a variety of microenvironmental signals, and not necessarily activated in a similar manner in the periphery and the thymic epithelium during clonal T cell selection. This implies that some PTM-containing epitopes may not be covered by central tolerance. Along similar lines, microenvironmental conditions that impose non-enzymatic PTMs (eg, an oxidative extracellular milieu) are common at sites of inflammation, ischemia or malignant progression, but not in the thymus.^92^ As a note, such stressful conditions can also lead to the generation of antigenic peptides derived from ‘cryptic’ translation (ie, from untranslated mRNAs).^93^ In support of the ability of healthy cells to drive ICD, at least in some settings, PTM-dependent epitopes have been attributed pathogenic value in some autoimmune disorders including diabetes and rheumatoid arthritis.^94 95^

The majority of human tumors are not driven by active viral infections. Nonetheless, like pathogen-infected dying cells, malignant cells can display a high antigenicity, largely reflecting the elevated mutational rate that frequently accompanies malignant transformation and disease progression in the context of immunoevasion.^96 97^ In a fraction of cases, such mutations prime immune responses as they affect coding regions of the genome. This is the case of non-synonymous point mutations (ie, mutations altering the amino acid sequence) as well as frameshift mutations caused by small insertions and deletions (indels) in proteins that are expressed and properly processed by the antigen presentation machinery, culminating in the exposure of tumor neoantigens (TNAs). TNAs exposed on the surface of malignant cells may have poor structural homology to self epitopes, hence partially resemble microbial epitopes and efficiently prime de novo immune responses.^75 98–101^ Some self antigens expressed by cancer cells can also initiate antitumor immunity. Because they are not unique to neoplastic tissues, but are also expressed by healthy or immunoprivileged tissues, such antigens have been designated tumor-associated antigens (TAAs). TAAs that have been shown to drive anticancer immunity, especially in the setting of therapeutic anticancer vaccination,^102 103^ encompass: (1) tissue differentiation antigens such as CD19, CD20, premelanosome protein (PMEL, best known as gp100), and melan-A (MLANA, best known as MART-1), as well as (2) ectopically expressed proteins such as carcinoembryonic antigens (CEAs), cancer/testis antigens, as well as multiple members of the MAGE and SSX protein families.^104–106^ Central tolerance against these antigens is leaky (implying that naïve T cell clone that express low-affinity TCRs are available) and peripheral tolerance can be overcome in the context of robust adjuvanticity.^106–108^ Thus, although TAAs are generally weaker at eliciting anticancer immunity as compared with TNAs,^109^ they can be relevant for ICD-driven immunity in tumors with low TNA load. Of note, the harsh conditions that characterize the tumor microenvironment (TME) and the extensive rewiring of signal transduction that characterizes malignant cells suggest that PTMs may play a predominant role in determining the antigenicity of cancer cells, a possibility that remains largely unexplored.^110^

Several factors influence the antigenicity of tumors evolving in immunocompetent, syngeneic hosts. First, the mutational burden (and thus the potential to generate TNAs) is heterogeneous across and within tumors, ranging from ~1 mutation/Mb in hematologic malignancies to >10 mutations/Mb in solid tumors with a hypermutant phenotype.^111–113^ Mutational burden and the TNA landscape also evolve over space (ie, in distinct tumor areas) and time (ie, at distinct stages of malignant progression) under the pressure of ongoing immunity and in response to increased genomic instability as well as chemotherapeutic or radiotherapeutic interventions,^97 114 115^ resulting in tumors or tumor areas with distinct antigenicity and hence differential ability to drive adaptive immune responses on RCD.^116^ That said, while mutational burden has been associated with the sensitivity of multiple tumors to ICBs^117–119^ and tumors with extensive immune infiltration (which often are genetically unstable) are characterized by a transcriptional signature of ICD,^120^ formal demonstration that mutational burden also influences the antineoplastic effects of ICD-based therapeutic regimens in the clinic is lacking. Preclinical evidence suggests that even cells with reduced mutational load can drive adaptive immunity,^121^ although the choice of the experimental model is expected to have a major role in this context (see *Detection of ICD in cancer*). Second, the antigenicity of malignant cells is directly related to antigen presentation, implying that genetic and epigenetic defects that compromise it can be beneficial for cancer cells.^122 123^ These defects, which are common in tumors with high mutational burden and robust T cell infiltration,^124 125^ include: (1) antigen loss and subclonal evolution, that is, the preferential expansion of cancer cell clones that do not express an antigen subjected to active immunity^114^ and (2) impaired antigen presentation as a consequence of mutations, deregulated expression, or structural alterations of key components of the antigen-presenting machinery including MHC Class I molecules, beta-2-microglobulin (B2M), transporter 1, ATP binding cassette subfamily B member (TAP1), TAP2 and proteasomal subunits.^125 126^

Of note, while the majority of ICD inducers are believed to have little impact on antigenicity and to operate by driving the correct spatiotemporal emission of DAMPs in the context of cell death (see *Sources of ICD adjuvanticity*), at least some ICD-triggering regimens may also boost antigenicity. This applies to potentially mutagenic agents, and to interventions that drive the reactivation of endogenous retroviruses and/or induce the expression of mutated genes or TAAs, such as CDK4/CDK6 inhibitors,^127^ ionizing radiation,^79 128^ DNA damage response (DDR) inhibitors,^129 130^ oncolytic viruses^131^ or HDAC inhibitors and other epigenetic regulators.^132–134^ Irrespective of this possibility, the ability of RCD to drive adaptive immunity intimately depends on the antigenicity of dying cells with respect to the availability and the functional status of the mature TCR repertoire of the host.

### Sources of ICD adjuvanticity

ICD driven by microbial pathogens is associated with arguably the most potent adjuvant signals for mammalian organisms, MAMPs. MAMPs encompass a variety of microbial products that operate both within infected cells and in their microenvironment to drive the recruitment and maturation of APCs, culminating with antigen (cross-)presentation to naïve T cells. MAMPs include microbial nucleic acid species (eg, viral single-stranded or double-stranded RNA or DNA, bacterial CpG-rich DNA) as well as structural components (eg, lipopolysaccharide, peptidoglycans, flagellin), and mostly mediate immunostimulatory effects via pattern recognition receptors (PRRs).^82 83^ The latter encompass numerous Toll-like receptors (TLRs) expressed on the cell surface and in endosomal compartments,^135–138^ as well as (1) cyclic GMP-AMP synthase (CGAS), a sensor of cytosolic double-stranded DNA (dsDNA)^139^; (2) RIG-I-like receptors (RLRs), a group of RNA-specific PRRs named after DExD/H-box helicase 58 (DDX58, best known as RIG-I)^140 141^; (3) NOD-like receptors (NLRs), a family of PRRs with broad ligand specificity named after a common nucleotide-binding oligomerization domain (NOD)^142 143^; (4) Z-DNA binding protein 1 (ZBP1), a nucleic acid sensor also known as DAI whose precise mechanism of activation remains unknown^144^; (5) heterogeneous nuclear ribonucleoprotein A2/B1 (HNRNPA2B1), a sensor of viral nuclear DNA and N6-methyladenosine-bearing RNAs.^145 146^ PRRs drive the production of numerous immunostimulatory that are key for pathogen control by the immune system.^147^

The same PRRs activated by MAMPs are also involved in the adjuvanticity of ICD induced in cancer cells by non-microbial stimuli.^68 148^ Thus, malignant cells exposed to a potentially immunogenic RCD inducer emit numerous DAMPs and cytokines that have been mechanistically linked to the initiation of adaptive immunity in preclinical models (table 1). These immunostimulatory molecules include (but most likely are not limited to): ATP,^149 150^ cellular nucleic acids,^151 152^ the non-histone, nuclear DNA-binding protein high mobility group box 1 (HMGB1),^153–156^ the member of the annexin superfamily annexin A1 (ANXA1),^157^ cytokines like type I interferon (IFN), C-C motif chemokine ligand 2 (CCL2), C-X-C motif chemokine ligand 1 (CXCL1) and CXCL10,^151 158 159^ ER chaperones like calreticulin (CALR), protein disulfide isomerase family A member 3 (PDIA3, also known as ERp57), heat shock protein family A (Hsp70) member 1A (HSPA1A, best known as HSP70), Hsp90 alpha family class A member 1 (HSP90AA1, best known as HSP90),^21 22 160^ cytosolic components like F-actin,^161^ and other mitochondrial products like DNA, reactive oxygen species (ROS), cardiolipin and transcription factor A, mitochondrial (TFAM).^36 162–164^ The major roles of ICD-associated DAMPs and cytokines are to: (1) enable the recruitment of APCs or their precursors to sites of RCD (eg, ATP)^149 150 165^, (2) spatially guide the interaction between APCs and dying cells (eg, ANXA1)^157^, (3) favor the phagocytosis of dying cells or their corpses (eg, CALR, ERp57, HSP70, HSP90, F-actin)^21 22 160 161^, (4) promote the maturation of APCs and their capacity to effect cross-presentation (eg, ATP, HMGB1, type I IFN and TFAM),^149 153 166–168^ and (5) facilitate the recruitment of T cells (eg, CCL2, CXCL1 and CXCL10).^151 158^ Of note, some emitted DAMPs are immunosuppressive (eg, adenosine),^169^ while others can switch to anti-inflammatory depending on the engaged PRR (eg, HMGB1), biochemical modifications such as oxidation (eg, HMGB1) or chronic release (eg, type I IFN).^53 70 170^ Indeed, while acute, robust inflammatory responses such as those driven by ICD ultimately engage anticancer immunity, indolent, chronic inflammation has consistently been associated with immunoevasion and tumor progression.^171^ Of note, the emission of DAMPs by dying cells occurs in the context of failing intracellular responses to stress, which relay danger signals to the rest of the organism for the preservation of systemic homeostasis.^172^ This implies that defects in multiple mechanisms that support cellular adaptation to stress may favor cell death, but at the same time may compromise the ability of dying cells to initiate adaptive immunity as a consequence of limited adjuvanticity.

**Table 1:** Major immunostimulatory DAMPs and cytokines mechanistically linked to ICD in cancer

Factor	Class	Effect	Main receptor(s)	Ref.
ANXA1	Surface protein	Directs APCs to dying cells	FPR1	157
ATP	Nucleotide	Promotes the recruitment, maturation and cross-presentation activity of APCs	P2RX7 P2RY2	149 150
CALR	ER chaperone	Promotes the uptake of dying cells and type I IFN secretion by APCs	LRP1	21 22 160 178 191
CCL2	Cytokine	Promotes T cell and neutrophil recruitment	CCR2	151 159
CXCL1	Cytokine	Promotes T cell and neutrophil recruitment	CXCR2	151 159
CXCL10	Cytokine	Promotes T cell and neutrophil recruitment	CXCR3	151 158 159
Cytosolic RNA	Nucleic acid	Promotes the secretion of type I IFN and other proinflammatory factors	MDA5 RIG-I TLR3	152
Cytosolic DNA	Nucleic acid	Promotes the secretion of type I IFN and other proinflammatory factors	AIM2 CGAS ZBP1	162 164
ERp57	ER chaperone	Promotes the uptake of dying cells by APCs	LRP1 (?)	160
Extracellular DNA	Nucleic acid	Promotes the recruitment and activation of neutrophils	TLR9	151 163
F-actin	Cytoskeletal component	Promotes the uptake of dying cells by APCs	CLEC9A	161
HMGB1	Nuclear DNA-binding protein	Promotes the maturation and cross-presentation activity of APCs	AGER TLR2 TLR4	153 154
HSP70	ER chaperone	Favors the uptake of dying cells by APCs	LRP1	21
HSP90	ER chaperone	Favors the uptake of dying cells by APCs	LRP1	21
TFAM	Transcription factor	Promotes APC maturation and recruitment	AGER	36
Type I IFN	Cytokine	Promotes APC maturation, cross-presentation, and T cell recruitment	IFNARs	158 194 195

AGER, advanced glycosylation end-product specific receptor; AIM2, absent in melanoma 2; ANXA1, annexin A1; APC, antigen-presenting cell; CALR, calreticulin; CCL, C-C motif chemokine ligand 2; CGAS, cyclic GMP-AMP synthase; CLEC9A, C-type lectin domain containing 9A; CXCL1, C-X-C motif chemokine ligand 1; CXCL10, C-X-C motif chemokine ligand 10; CXCR2, C-X-C motif chemokine receptor 2; CXCR3, C-X-C motif chemokine receptor 3; DAMP, danger-associated molecular pattern; ER, endoplasmic reticulum; FPR1, formyl peptide receptor 1; HMGB1, high mobility group box 1; HSP, heat shock protein; ICD, immunogenic cell death; IFN, interferon; IFNAR, interferon-alpha/beta receptor; LRP1, LDL receptor-related protein 1; P2RY2, purinergic receptor P2Y2; P2X7, purinergic receptor P2X 7; TFAM, transcription factor A, mitochondrial; TLR2, Toll-like receptor 2; TLR3, toll like receptor 3; TLR4, toll like receptor 4; TLR9, toll like receptor 9; ZBP1, Z-DNA binding protein 1.

Macroautophagy (herein referred to as autophagy) is a cytoprotective lysosomal pathway for the degradation of superfluous or potentially dangerous cytosolic material and organelles.^173–176^ Robust evidence indicates that autophagy is required for the preservation of lysosomal ATP stores in the course of most (but not all) variants of ICD.^149 166^ As a consequence of cellular blebbing, which is mediated by lysosomal-associated membrane protein 1 (LAMP1) and pannexin 1 (PANX1), ATP is released into the extracellular space.^165 177^ As an alternative, the ICD-associated release of ATP can occur through anterograde ER-to-Golgi transport.^178^ Extracellular ATP exerts its adjuvanticity by binding to purinergic receptor P2Y2 (P2RY2), which favors the recruitment of APCs and their precursors, and purinergic receptor P2X7 (P2RX7), which favors their activation and consequent release of the immunostimulatory cytokine interleukin (IL)−1β.^149 150 179^

The integrated stress response (ISR), a multipronged molecular mechanism for the preservation of cellular homeostasis is required for the exposure of ER chaperones on the cell surface during ICD.^46 180–182^ In particular, ER stress induced by anthracyclines stimulates the inactivating phosphorylation of eukaryotic translation initiation factor 2 subunit alpha (EIF2S1, best known as eIF2α) by eukaryotic translation initiation factor 2 alpha kinase 3 (EIF2AK3, best known as PERK),^183^ culminating in the CASP8- and B cell receptor associated protein 31 (BCAP31)-dependent translocation of ER chaperones to the outer leaflet of the plasma membrane.^160 178 180^ For most ICD inducers, the entire process also relies on anterograde ER-to-Golgi transport mediated by vesicle-associated membrane protein 1 (VAMP1) and synaptosomal-associated protein 25 (SNAP25)^53 180 184^ and requires the concomitant production of ROS.^71^ Of note, the ICD-associated exposure of some ER chaperones (notably, CALR) on the cell surface appears to be regulated by C-X-C motif chemokine ligand 8 (CXCL8),^184^ ER Ca^2+^ levels,^185^ as well as CASP2, long non-coding RNAs (eg, ncRNA-RB1 and miR-27a), and plasma membrane integrins, at least in some settings.^186–189^ Surface-exposed CALR (and other ER chaperons) promotes the uptake of dying cells or their corpses by APCs, at least in some settings on interaction with LDL receptor related protein 1 (LRP1).^178 190^ Moreover, CALR exposure appears to drive type I IFN secretion by APCs,^191 192^ which is also expected to contribute to the immunogenicity of RCD.

The mechanisms for the preservation of cellular homeostasis in response to infection are also intimately involved in the adjuvanticity of ICD, even when the latter is not driven by microbes.^193^ Indeed, multiple nucleic acids of endogenous derivation can be detected by PRRs to initiate danger signalling, generally based on ectopic localization or structural modifications that arise during stress responses.^135^ Thus, chemotherapy-driven ICD involves the activation of TLR3 by endogenous RNA species, resulting in type I IFN secretion and the consequent initiation of an autocrine loop that culminates with CXCL10 release.^158^ Along similar lines, cancer cells succumb to ionizing radiation as they produce type I IFN downstream of CGAS signalling driven by cytosolic dsDNA.^194 195^ By binding to its cognate receptor, type I IFN mediates robust immunostimulatory effects on both APCs and effector cells.^196^ It also triggers the production of IFN-stimulated genes (ISGs) like CXCL10. CXCL10 then acts as a chemoattractant for T cells and, together with CXCL1 and CCL2, for neutrophils, which (at least in some settings) appear to contribute to the ICD-driven killing of residual cancer cells in an antigen-independent fashion.^151 158^ Of note, cancer cell-derived nucleic acids can also mediate immunostimulatory effects by driving type I IFN secretion in APCs, generally on the transfer of nucleic acid-containing extracellular microvesicles between these two cellular compartments.^195 197^

The precise stress responses involved in the release of other ICD-associated DAMPs including ANXA1 and HMGB1 remain to be fully elucidated. Irrespective of this unknown, ANXA1 is known to spatially direct APCs to dying cells on interaction with formyl peptide receptor 1 (FPR1),^157^ while HMGB1 can mediate robust immunostimulatory functions by signaling via advanced glycosylation end-product specific receptor (AGER, also known as RAGE) and TLR4,^153 154 167 198–200^ an activity that appears to depend on oxidation status.^201 202^ Moreover, it has become clear that the molecular machinery involved in ICD-associated DAMP emission exhibits some degree of variation depending on ICD inducer and cell type. Thus, CALR exposure and ATP secretion are required for the full-blown immunogenicity of cancer cells succumbing to hypericin-based PDT, but this can occur independently of eIF2α phosphorylation and autophagy activation.^178 203^ Likewise, neoplastic cells succumbing to necroptosis-driven ICD release ATP and HMGB1, but CALR is exposed at low levels on the plasma membrane and ISR activation appears to be dispensable.^59 204^

The critical role of DAMP signaling in the immunogenicity of RCD has been established by a plethora of complementary mechanistic experiments aimed at: (1) the pharmacological or genetic inhibition of the signaling pathways involved in DAMP release (eg, with cancer cells expressing a non-phosphorylatable variant of eIF2α)^180^; (2) the artificial activation of stress responses responsible for DAMP emission (eg, with ER stressors in neoplastic cells undergoing a variant of RCD otherwise not associated with activation of the ISR)^205 206^; (3) the knockout or knockdown of DAMP-coding genes (eg, with cancer cells depleted of HMGB1 by RNA interference)^23 153^; (4) DAMP neutralization/blockade (eg, with cells overexpressing an intracellular enzyme degrading cytosolic dsDNA)^194^; (5) the exogenous complementation of DAMPs (eg, with recombinant CALR administered to cells undergoing RCD in the absence of CALR exposure)^22 180 207 208^; (6) the upregulation of antagonistic processes (eg, with cancer cells overexpressing the antiphagocytic molecule CD47)^209 210^; (7) the blockade of DAMP receptors (eg, with monoclonal antibodies specific for type I IFN receptors)^158^ and (8) the knockout or knockdown of genes encoding for DAMP receptors in the host (eg, with *Fpr1^-/-^* mice).^157^ Several lines of clinical evidence also suggest that proficient danger signaling is critical for cancer patients to obtain clinical benefits from ICD-inducing therapies.^211^ These findings generally relate to the prognostic or predictive value of (1) activation of stress responses impinging on DAMP emission in cancer cells (eg, eIF2α phosphorylation in association with high CALR levels in biopsies from patients with non-small cell lung cancer)^212^; (2) the expression levels of specific DAMPs (eg, HMGB1 levels in biopsies from patients with breast cancer subjected to adjuvant anthracycline-based chemotherapy)^213 214^; (3) DAMP emission by cancer cells (eg, CALR exposure on blasts in patients with acute myeloid leukemia)^215 216^; (4) actual danger signaling in the TME (eg, gene signatures of type I IFN signaling in subjects with breast cancer)^217^; (5) loss-of-function polymorphisms in genes encoding DAMP receptors (eg, *P2R×* *7*, *TLR4* and *FPR1* polymorphisms in patients with breast carcinoma receiving neoadjuvant anthracyclines)^23 150 153 157^ and (6) the expression levels of DAMP antagonists (eg, CD47 expression on cancer cells in patients with acute myeloid leukemia, esophageal squamous cell carcinoma and ovarian clear cell carcinoma).^218–220^ These are only a few examples corroborating the relevance of DAMP signaling for RCD to be sensed as immunogenic in patients.

### Microenvironmental factors influencing ICD

Although some tissues respond to pathogenic infection more robustly than others (reflecting the differential abundance of tissue-resident APCs), cells succumbing to microbial infection generally drive adaptive immunity irrespective of anatomical location.^221^ Conversely, the microenvironment of dying cancer cells is a major determinant of their ability to initiate adaptive immune responses, even in the presence of sufficient antigenicity and adjuvanticity,^5 222^ and this has major implications for the choice of experimental models for the assessment of ICD in vivo (see *In vivo models*).

There are several mechanisms whereby the microenvironment of developing tumors can antagonize the initiation or execution of ICD, largely reflecting the ability of various neoplasms to establish peripheral tolerance. So-called ‘cold’ and ‘excluded’ tumors are poorly infiltrated by immune cells including APCs and their precursors at baseline, implying that the likelihood for dying cancer cells and their corpses to be productively processed and drive cross-priming is reduced.^223 224^ Priming is also limited by coinhibitory receptors expressed by tumor-infiltrating T cells including CTL-associated protein 4 (CTLA4) and hepatitis A virus cellular receptor 2 (HAVCR2, best known as TIM-3), a glycoprotein that binds to HMGB1 as well as the ‘eat me’ signal phosphatidylserine on the surface of dying cells.^152 225^

Moreover, the activity of APCs that infiltrate malignant lesions is generally inhibited by immunosuppressive cytokines including (but not limited to) IL-10 and transforming growth factor beta 1 (TGFB1).^226 227^ These bioactive factors are abundantly produced in response to hypoxia and during chronic inflammation, and are robustly associated with immunoevasion and tumor progression.^228^ IL-10 and TGFB1 are secreted by cancer cells and by immunosuppressive immune cells actively recruited to the TME, such as CD4^+^CD25^+^FOXP3^+^ regulatory T (T_REG_) cells, M2-polarized tumor-associated macrophages (TAMs), and/or myeloid-derived suppressor cells (MDSCs).^229–231^ Importantly, these immune cell populations express high levels of ectonucleoside triphosphate diphosphohydrolase 1 (ENTPD1, best known as CD39) and 5'-nucleotidase ecto (NT5E, best known as CD73),^232–234^ two enzymes that cooperate to convert extracellular ATP into adenosine, which also mediates robust immunosuppressive effects.^235^ Thus, T_REG_ cells, M2-polarized TAMs and MDSCs also have direct ICD antagonizing effects.

The redox status of the TME and individual DAMPs or their receptors may also affect the ability of RCD to drive adaptive anticancer immunity. For example, the release of oxidized HMGB1 by cancer cells undergoing pyroptosis, a gasdermin-dependent form of RCD generally associated with inflammasome activation,^1^ limits anticancer immunity as it favors the expression of coinhibitory ligands.^236^ In contrast, oxidized mitochondrial DNA favors inflammasome activation and hence the secretion of immunostimulatory factors such as IL-1β in the TME,^237^ although the actual pathologic relevance of this pathway remains unknown.

Another major mechanism for progressing tumors to evade ICD at the execution phase (ie, the ability of ICD-driven CTLs to mediate cytotoxic effects) relies on immune exhaustion, that is, the establishment of dysfunction in tumor-infiltrating T cells.^238–241^ Coinhibitory receptors including programmed cell death 1 (PDCD1, also known as PD-1) are major (but not the sole) players in this setting. Indeed, activated CTLs have elevated metabolic demands, and both glucose and amino acids are generally limited in the TME.^242 243^ Moreover, several immunosuppressive metabolites that are enriched in the TME besides adenosine, such as kynurenine and lactate,^244 245^ and cytokine-dependent immunosuppression also operate on CTLs.^224 246 247^ Finally, vascular defects and the dense stromal reaction that characterize some tumors (eg, pancreatic cancer) can constitute a physical barrier to tumor infiltration by CTLs cross-primed in tumor-draining lymph nodes,^248 249^ de facto hampering the execution phase of ICD. Besides exemplifying the critical importance of local microenvironment for the immunogenicity of RCD, these observations explain why the same cancer cells receiving the same ICD inducers in vitro and in vivo may differ in their ability to initiate adaptive immunity.

Importantly, multiple ICD-inducing therapeutics as well as various therapies that do not promote ICD can mediate immunomodulatory effects on the TME by directly interacting with immune cell populations (rather than with cancer cells).^73^ Although such immunomodulatory effects are important for the ultimate efficacy of therapy in patients, they are conceptually and mechanistically unrelated to ICD induction, and hence will not be discussed further here.

### Detection of ICD in cancer

Over the past two decades, an intensive wave of investigation has unveiled major mechanistic and correlative aspects of ICD as a process culminating in the activation of adaptive immunity against dying cells. Experimental strategies conceived to assess the immunogenicity of RCD encompass: (1) the study of DAMP emission from (and activation of relevant stress responses in) dying cells; (2) biochemical and functional tests to assess the activation of APCs and their ability to mediate cross-priming, in vitro and (3) the ability of dying cells to initiate adaptive immunity in vivo, in immunocompetent syngeneic hosts. Here, we will summarize current methods to assess ICD in oncological settings.

#### In vitro assays with cancer cells

Robust experimental evidence indicates that the ability of RCD to promote adaptive immunity critically relies on a progressing (but not exhausted) CTL response.^121^ Presumably, this reflects the need for the availability of one or more TNAs/TAAs above a specific threshold, coupled to the timely delivery of danger signals (which are likely to vary depending on RCD inducer and tumor cell type). Thus, while mouse cancer cells exposed to cardiac glycosides release ICD-associated DAMPs, they are unable to initiate protective anticancer immunity once inoculated in immunocompetent syngeneic hosts as cytotoxicity is limited and tumors develop at the vaccination sites.^250^ Similarly, repeated freeze-thawing causes rapid cell death coupled to massive TNA/TAA release, but mouse cancer cells subjected to this harsh procedure cannot be used to protect syngeneic immunocompetent hosts against a subsequent challenge with living cells of the same type, most likely due to suboptimal adjuvanticity.^20 59^ The cytotoxic response driven by ICD inducers has classically been assessed by terminal cell death biomarkers such as plasma membrane permeabilization, as well as by conventional biomarkers of apoptosis, including phosphatidylserine exposure, mitochondrial transmembrane potential dissipation and initiator or effector caspase activation.^148^ The rationale and principles of these assays have been extensively described by us and others,^58 251 252^ and hence will not be discussed further here. That said, the existence of ICD variants relying on the necroptotic machinery and/or occurring independent of caspase activation^1 59 61^ identifies a need for tools to measure ICD induction that can accommodate all potentially relevant RCD pathways.

Intracellular responses to stress mechanistically involved in DAMP release have also been employed as surrogate ICD biomarkers. ISR activation has generally been monitored in its three reticular components by the assessment of eIF2α phosphorylation, X-box binding protein 1 (XBP1) splicing and activating transcription factor 6 (ATF6) nuclear translocation, in some instances along with heat shock protein family A (Hsp70) member 5 (HSPA5, also known as GRP78) upregulation. These biomarkers are detected by immunoblotting, flow cytometry, immunofluorescence microscopy, immunohistochemistry and/or RT-PCR, using dedicated antibodies or probes.^33 183 253^ ICD-associated PRR activation in cancer cells has been classically measured by immunoblotting with antibodies specific for key phosphorylated transducers, such as phosphorylated IFN regulatory factor 3 (IRF3) downstream of CGAS signaling,^254 255^ or with transcription-based luminescent reporters.^254 256–258^ As an alternative approach, the ectopic accumulation of nucleic acids has been monitored, generally by immunofluorescence microscopy with dedicated antibodies or by subcellular fractionation coupled to optional enzymatic digestion of nucleic acids and absorbance-based quantification.^158 256^ Activation of autophagy in cells exposed to potential ICD inducers has largely been monitored by the concomitant assessment of microtubule associated protein 1 light chain 3 (MAP1LC3, best known as LC3) lipidation and degradation of an autophagic substrate such as sequestosome 1 (SQSTM1, also known as p62) by immunoblotting, in the context of appropriate control conditions.^259^ Autophagy is indeed a dynamic process and the mere analysis of LC3 lipidation in cells responding to stress conditions cannot be employed to discriminate between autophagy activation and inhibition.^259^

Cell surface exposure of ER chaperones as a surrogate biomarker of ICD can be determined by (1) flow cytometry in non-permeabilized early apoptotic cells using specific antibodies or dedicated constructs (eg, CALR fused to the HaloTag) combined with vital dyes like 7-aminoactinomycin D (7-AAD), 4′,6-diamidino-2-phenylindole (DAPI) or propidium iodide (PI), which enable the exclusion of dead cells from the analysis^41 180 205 260^; (2) fluorescence microscopy, in cells fixed with low concentration of paraformaldehyde and then immunostained with antibodies specific for ER chaperones^21 32 43 160 261^; (3) fluorescence microscopy or video microscopy, in cells constitutively expressing a construct in which specific the ER chaperone of choice is fused to GFP or another fluorescent tag^33 205 250 262^ and (4) immunoblotting, in cells previously subjected to cell surface protein biotinylation followed by streptavidin-mediated precipitation.^160 263^

Two main approaches have been harnessed to determine the release of ICD-associated soluble DAMPs and cytokine from dying cells: (1) DAMP/cytokine quantification in culture supernatants and (2) quantification of residual DAMPs/cytokines in the intracellular microenvironment. Of note, while the former approach is universally applicable, the latter cannot be employed for DAMPs and cytokines that are actively synthesized before release, such as type I IFN and CXCL10.^148^ Thus, extracellular and intracellular ATP can be quantified with commercial luminescence-based assays on the culture supernatants and cell lysates, respectively.^149 150 178 264^ Extracellular ATP and its degradation products (ADP, AMP and adenosine) can also be quantified by targeted mass spectrometry,^149^ while its intracellular counterpart can be monitored by flow cytometry or fluorescence microscopy on staining with the ATP-specific dye quinacrine or implementation of ATP-specific fluorescence resonance energy transfer (FRET)-based reporters.^165 265^ Finally, ANXA1, HMGB1, type I IFN and CXCL10 secretion by cells undergoing RCD have been classically monitored by commercial ELISA or immunoblotting on culture supernatants or cell lysates.^32 151 153 157 158 266^ Moreover, a fluorescent version of HGMB1 is available that enables the assessment of HMGB1 release by fluorescence microscopy or video microscopy, as a function of residual cell fluorescence.^33 42 250 267^ Of note, while RT-PCR is commonly employed to monitor type I IFN and CXCL10,^158 268^ this approach de facto measures PRR signaling, as transcription is not necessarily coupled with translation and secretion.

#### In vitro assays with immune cells

While intracellular responses to stress and DAMP emission can be equally monitored in mouse and human tumor models, in vivo studies can currently be performed only in the former (see *In vivo models*). To partially circumvent this issue and enable a functional assessment of the immunogenicity of RCD in both the mouse and human system, the field has borrowed multiple classical assays from immunology. In general, these experiments aim at evaluating whether dying cancer cells can stimulate the ability of APCs to optimally cross-prime CTLs and hence initiate an adaptive immune response.

In particular, human or mouse APCs exposed to human or mouse dying cancer cells, respectively, are often investigated for: (1) their ability to engulf dying cells or their corpses; (2) their maturation status and migratory capacity and (3) their ability to cross-present antigenic material to CTLs. Phagocytosis is often assessed by coculturing APCs or their precursors and dying cancer cells on individual prelabeling of both compartments with distinct non-toxic fluorescent dyes that remain in the cytoplasm, such as carboxyfluorescein succinimidyl ester (CFSE) or PKH26, followed by fluorescence microscopy or flow cytometry.^32 269 270^ As an alternative, only cancer cells are prelabeled, and phagocytosis is monitored on staining the coculture with monoclonal antibodies specific for the APC of choice.^264 266^ Moreover, apoptotic cell uptake has been measured by injecting pre-labeled dying mouse cancer cells intravenous, followed by splenocyte isolation and flow cytometry.^268 270^ The maturation status of APCs has been classically measured by flow cytometry, on staining of cell cocultures with antibodies specific for MHC class II molecules and costimulatory molecules including CD80, CD83 and CD86 (which are all upregulated during maturation).^21 43 263 271^ As an alternative or complementary approach, functional maturation has been monitored by the detection of cytokines secreted in culture supernatants by APCs acquiring an immunostimulatory phenotype, including (but not limited to) IL-1β, IL-6, IL-12 and IL-23.^41 150 263^ ELISA and flow cytometry on intracellular staining with dedicated antibodies remain the techniques of choice for the latter strategy. That said, the intracellular assessment of IL-1β requires an antibody directed against the fully mature variant of the protein, as its precursor is not secreted.^272 273^ Migratory capacity (which reflects the ability of dying cells to secrete chemotactic factors) has been evaluated by transwell assays^274 275^ or by dedicated microfluidic devices that allow for video microscopy (if cells are prelabeled with fluorescent dyes).^157 276^

The cross-priming potential of APCs exposed to cancer cells undergoing RCD has most frequently been assessed by coculturing them with syngeneic, naïve T cells, followed by the assessment of: (1) proliferative T cell response, generally by flow cytometry on previous labeling with CFSE^271 277^; (2) T cell activation status, most often by flow cytometry on staining with monoclonal antibodies specific for surface proteins linked to activation (eg, CD69, LAMP1, PD-1)^150 153 278^; (3) T cell functional profile, either by flow cytometry on intracellular staining with monoclonal antibodies specific for effector molecules such as IFN-γ, perforin 1 (PRF1) and granzyme B (GZMB) or by quantification of extracellular IFN-γ by ELISPOT.^21 41 268 278^ Finally, cytotoxic T cell functions on cross-priming can be tested by measuring the lysis of living cancer cells of the same type of those employed for APC pulsing^46 279 280^ or by analyzing the response to specific TAAs.^281 282^

#### In vivo models

**Figure 2:**
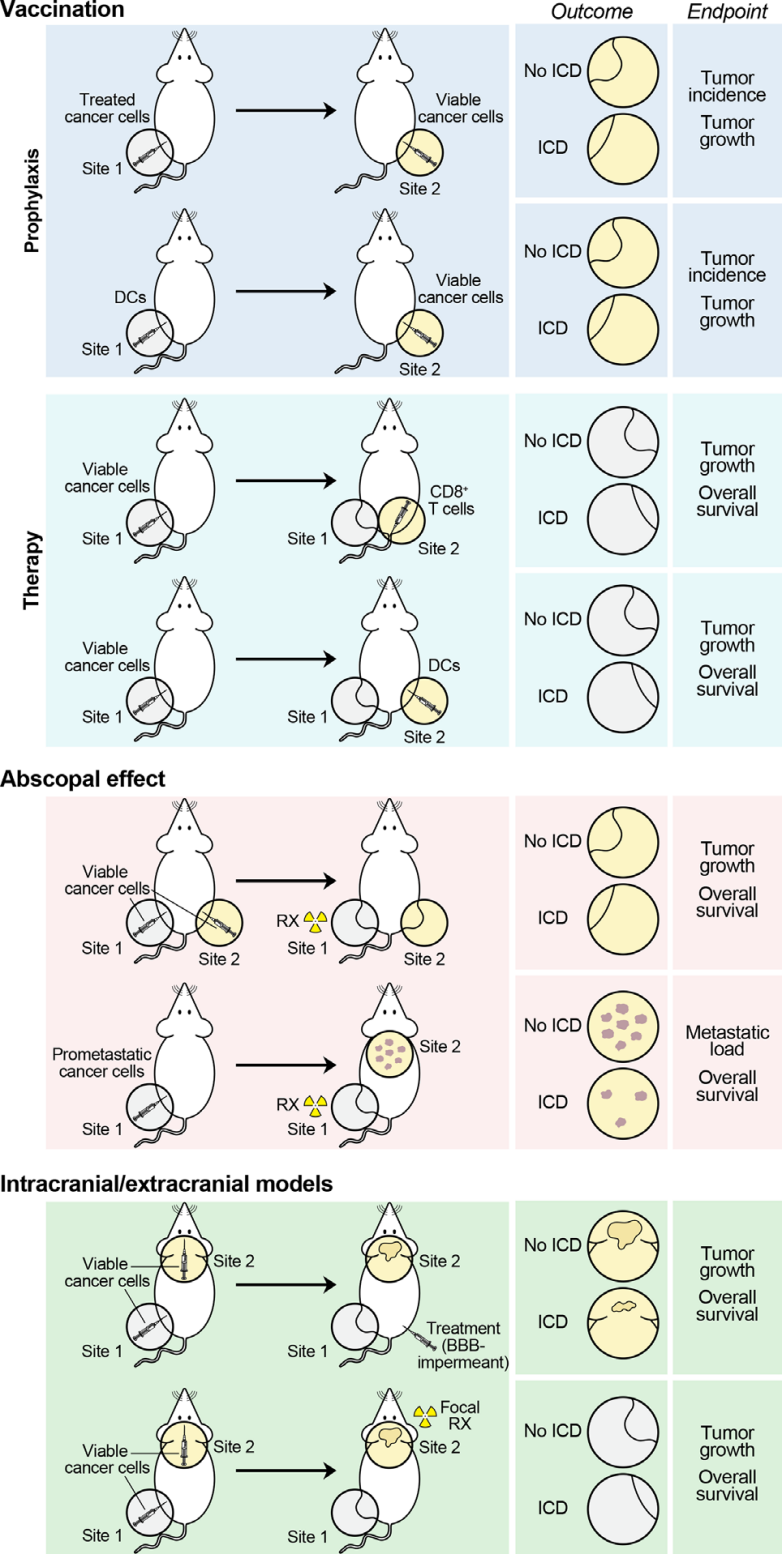
Current methods to assess ICD in vivo, in oncological settings. Current models to ascertain the ability of dying cancer cells to elicit an adaptive, tumor-specific immune response in vivo invariably rely on mouse neoplasms established in immunocompetent syngeneic hosts. In prophylactic models, mouse cancer cells succumbing in vitro to a potential inducer of immunogenic celldeath (ICD) are used as a vaccine, either as such, or on loading on immature, syngeneic dendritic cells (DCs). The ability of mice to reject (tumor incidence) or control (tumor growth) a rechallenge with living cancer cells of the same type inoculated 1–2 weeks later is monitored as a sign of protective anticancer immunity. In therapeutic settings, mouse tumors developing in immunocompetent syngeneic hosts are treated with autologous DCs preloaded with cancer cells exposed to a potential ICD inducer in vitro (generally in combination with immunological adjuvants), or with autologous CD8^+^ cytotoxic lymphocytes primed in vitro by the same DCs (generally in combination with IL-2 or other cytokines that support expansion in vivo). Tumor control and mouse survival are monitored as indicators of therapeutic anticancer immunity. In abscopal models, mouse cancer cells are harnessed to generate lesions at distant anatomical sites (either artificially, or exploiting the natural capacity of some cell lines to generate metastases), followed by treatment at only one disease site (generally in the context of otherwise inactive systemic immunostimulation). Tumor control at the non-treated disease site and mouse survival are monitored as signs of systemic anticancer immunity with therapeutic relevance. Finally, in intracranial/extracranial models, mouse cancer cells are employed to generate one intracranial and one extracranial tumor, only one of which receives treatment (generally, a systemic agent that cannot cross the blood–brain barrier [BBB] for extracranial lesion, or radiation therapy for intracranial lesions, in both cases in combination with otherwise inactive immunostimulants). As in abscopal models, tumor control at the non-treated disease site and mouse survival are monitored as indicators of therapeutic anticancer immunity with systemic outreach. In all these models, mice achieving systemic, long-term disease eradication are often rechallenged with cancer cells to monitor durability (with the same cancer cells employed for disease establishment) and specificity (with unrelated, but syngeneic cancer cells). ICD, immunogenic cell death; IL-2, interleukin 2.

Only a few models are currently available to investigate ICD in vivo (figure 2). The gold standard approach to assess the ability of dying cells to initiate adaptive immunity involves vaccination assays with immunocompetent, syngeneic mice.^148^ In this context, mouse cancer cells are exposed to a potential ICD inducer in vitro and then administered as a vaccine *s.c*., in the absence of any immunological adjuvant and on the removal of exogenous chemical entities (if any, such as the ICD inducer itself). One to two weeks later, mice are challenged *s.c*. with living cancer cells of the same type (at the minimal dose that is 100% effective at generating progressing lesions in naïve mice) and followed over 40–60 days for tumor incidence and growth.^20 22 153 178 208^ Not only the percentage of tumor-free mice, but also the growth rate of tumors potentially developing despite a vaccine-induced adaptive immune response are usually employed as indicators of (at least some) degree of immunogenicity. Specificity is confirmed by re-challenging tumor-free mice at the end of the experiment with another syngeneic cancer cell line, which is expected to generate progressing neoplastic lesions in 100% of mice. Of note, vaccination can also be performed with APCs exposed to dying cancer cells in vitro or implemented in therapeutic (rather than prophylactic) settings, that is, as a treatment of established tumors.^157 283–285^ Moreover, CD8^+^ T cells cross-primed in vitro by APCs exposed to cancer cells undergoing ICD have been employed in adoptive transfer experiments to treat tumors previously established with the living cancer cells of the same type.^32 286^ Importantly, comparing the efficacy of any RCD inducer against mouse cancer cells growing in immunocompetent, syngeneic versus immunodeficient mice can provide hints on the ability of such intervention to drive ICD (in such case, therapeutic efficacy will be limited in immunodeficient hosts). However, this latter experimental setting is intrinsically unsuitable to discriminate between ICD induction and ICD-unrelated immunostimulation (see *Interpretation of* *ICD*).

Three alternative approaches to assess ICD in vivo, in immunocompetent syngeneic systems, critically rely on the measurement of tumor growth at non-treated disease sites, which implies they can only be implemented with localized therapies (eg, focal ionizing radiation and intratumoral delivery of therapeutic agents that fail to reach active concentrations systemically)^79 287–289^ or when the non-treated site is biologically inaccessible to treatment but accessible to CTLs (eg, brain metastases in a host receiving chemotherapeutics that do not cross the blood-brain barrier).^290^ Models of the so-called ‘abscopal response’, that is, the regression of an out-of-field lesion in patients receiving ionizing radiation to a distant disease site,^291^ have proven highly instrumental in this setting. Usually, immunocompetent mice are grafted with either (1) cancer cells to generate two slightly asynchronous lesions *s.c*., at anatomically distant sites or (2) metastasis-prone cancer cells to generate a palpable lesion *s.c*. and metastatic (lung) dissemination.^79 288 292^ In both scenarios, only one of the subcutaneous lesion(s) receives ionizing radiation (generally in the presence of an immunostimulatory molecule that has no single-agent systemic effect), and the response of non-irradiated lesion, metastatic load and overall survival are monitored as indicators of ICD induction coupled to activation of adaptive immunity with systemic outreach.^78 79 287 288 292 293^ Mice rejecting irradiated and non-irradiated lesions can be rechallenged 30–40 days after disease eradication with the same cancer cells employed originally to assess the durability of protection, as well as with syngeneic cancer cells of another type to monitor its specificity.^49 292 293^ Likewise, distinct cell types can be employed for the generation of primary and secondary lesions to verify that the in situ vaccination effect generated by ICD is antigen specific.^294^ Finally, models of simultaneous intracranial and extracranial disease have been harnessed to monitor the ability of systemic chemotherapeutic agents that are unable to penetrate the blood–brain barrier and focal ionizing radiation to elicit anticancer immunity in the periphery.^295^ Of note, in these latter models, the presence of extracranial lesions increases the efficacy of immunotherapy against cranial neoplasms by stimulating CTL trafficking,^296^ although the relevance of this phenomenon for ICD-driven immunity remains to be ascertained.

All these models are amenable to ex vivo studies aimed at: (1) the characterization of DAMPs released by cancer cells responding to stress in situ; (2) the immunological profiling of APCs and CTLs underlying the initiation and execution of anticancer immunity in vivo and (3) the identification of mechanistic vs correlative aspects of ICD elicited in vivo by the stressor(s) of choice (eg, with depletion, blockage or neutralization strategies).^79 287 288 293^

### Interpretation of ICD

Most of the assays that are currently available to estimate the propensity of RCD to drive adaptive immune responses fail to take into proper consideration the complexity that is inherent to ICD, which (as detailed above) ultimately represents a highly contextual process that depends on (1) initiating stimulus, (2) responding cell and (3) the host (figure 1). In this context, the interpretation of ICD-related assays faces a number of challenges that should be always kept under consideration.

Surrogate biomarkers of ICD, such as the accumulation of cytosolic DNA, the exposure of ER chaperones on the cell surface or the release of ATP and/or HMGB1 by dying cells, as well as biomarkers of the stress responses that underlie their emission, have been instrumental for the characterization of the key molecular players of process, and the identification of potential ICD inducers in large screening efforts.^183 184 262^ However, not all triggers of ICD operate via the same molecular mechanisms, as demonstrated by the ability of hypericin-based PDT to initiate anticancer immunity irrespective of the autophagic proficiency of malignant cells.^203^ Moreover, the emission of ICD-associated DAMPs according to the correct spatiotemporal pattern is required, but not sufficient, for APCs to initiate CTL-dependent immune responses against dying cells. Thus, cardiac glycosides trigger a multipronged stress response culminating with all major surrogate biomarkers of ICD, and yet cannot establish protective immunity in gold-standard vaccination assays.^250^ These observations suggest that the ability of any stressor to drive a variant of RCD associated with adaptive immunity cannot be extrapolated by in vitro assays focusing on cancer cells.

Although their scalability to screening applications is limited, immunological assays testing the ability of APCs primed with dying cells to engage in the sequential process leading to cross-priming (which is an absolute requirement for adaptive immunity in this setting) obviously offer a more precise assessment of the immunogenicity of RCD. However, these assays are also inherently limited in that they are unable to assess two major prerequisites for cross-priming: (1) the ability of APCs to physically reach sites of RCD and (2) the existence of naïve T cell clones specific for antigens expressed by dying cells, ultimately calling for validation with in vivo models.

Vaccination assays have been highly instrumental for the demonstration that syngeneic dying cells can drive adaptive immunity in the presence of adequate antigenicity and adjuvanticity.^20 22 153 178 208 297^ However, the use of tumor-naïve hosts enables a very high degree of sensitivity, which may not necessarily be advantageous if clinical applications are the ultimate goal. In this context, abscopal and intracranial/extracranial models may offer increased pathophysiological relevance, as (with the limitations described above) they mimic established metastatic disease in humans. However, these models are limited in that they can only measure systemic immunity to local therapies or agents that do not penetrate the blood–brain barrier.^79 288^ Moreover, largely reflecting the clinical scenario, eliciting systemic immunity with disease-eradicating potential in these models is challenging, and often requires the provision of additional immunostimulatory signals that antagonize peripheral tolerance.^77–79^ Such a limited sensitivity (although clinically relevant) may be detrimental at early stages of discovery when optimal ICD conditions are yet to be determined.

As a group, ICD-relevant in vivo models are affected by multiple issues including the fact that only murine systems can be investigated, and it cannot be excluded that the molecules and cell populations at play in the human setting may be different (at least to some degree).^298 299^ Moreover, all current models for the detection of ICD in vivo rely on the establishment of primary lesions with murine cancer cell lines, which (1) have been immunoedited and acquired the ability to evade immunosurveillance in their original host (implying that they do not properly recapitulate primary oncogenesis), (2) have a relatively low and homogeneous mutational burden compared with human disease (and hence fail to recapitulate the mutational and antigenic landscape of human tumors), (3) generally do not establish a complex stromal and endothelial architecture as a consequence of their elevated proliferation rate (which differs from human neoplasms) and (4) are implanted by injection, which per se may mediate at least some degree of immunostimulation.^300–303^ Finally, the need for fully syngeneic settings (to prevent rejection based on HLA mismatch) restricts remarkably the spectrum of cell lines and rodent strains that can be harnessed for this purpose, with a predominance of models based on the C57BL/6 or BALB/c background.^304^

Current efforts to circumvent, at least in part, these issues involve the use of orthotopic models (which offer improved microenvironmental features), transgene-driven models (which may offer a superior view on early oncogenesis) and carcinogen-driven models (which have superior heterogeneity).^305–307^ However, not all of these systems are compatible with vaccination and/or abscopal assays, implying that ICD induction can only be imprecisely addressed by complementing in vitro observations with therapeutic efficacy in immunocompetent vs immunodeficient animals. Moreover, considerable efforts are being devoted to the development of humanized mice, which ultimately may enable the assessment of ICD induction in vivo with human cancer cells.^308–310^ Most often, these models involve the engraftment of functional human immune cells from various sources into highly immunodeficient mice as a means to (partially and temporarily) reconstitute a functional human immune system.^304^ The major limitations of this approach (which vary in severity depending on the precise experimental protocol) include: (1) the ability of human immune cells to react against their mouse counterparts due to cross-species incompatibility and consequently graft-versus-host disease^311 312^; (2) the limited ability of (at least some) mouse cytokines to support immune cell reconstitution and function via human cytokine receptors^313^; (3) the hitherto poorly understood cross-talk between residual components of the mouse immune system (eg, macrophages, granulocytes, endothelial cells) and reconstituted human cells^309^ and (4) the lack of thymic selection.^314^ Although refined strategies to circumvent these issues are being investigated, including the use of mice engineered to express human cytokines as well as the coimplantation of thymic fragments,^313 315–320^ this technology is hitherto immature for the assessment of ICD in the human system.

In summary, the assessment of ICD requires experimental support from a variety of in vitro and in vivo assays that should cumulatively assess the ability of malignant cells undergoing RCD at the natural anatomical location to recruit APCs and stimulate them to initiate adaptive anticancer immunity.

### Conclusions and perspectives

Accumulating evidence demonstrates that the initiation of ICD is critical for the elimination of infectious pathogens and stands out as a major therapeutic goal for cancer therapy, especially in consideration of the current clinical success of ICBs.^321 322^ Indeed, the ability of several agents to drive ICD in oncological settings is hampered by the robust immunosuppressive circuitries established in the TME during the tumor-host coevolution, and ICBs may be instrumental for the inactivation of such circuitries.^56 77 79 323 324^

There are three major obstacles precluding the full clinical potential of ICD inducers to be realized. First, most of these agents have been developed clinically based on the paradigm of maximum tolerated dose (MTD), and it is now clear that greater cytotoxicity does not necessarily coincide with optimal immunogenicity.^194^ Moreover, the vast majority of clinically employed ICD inducers have been developed preclinically in immunodeficient models of disease, implying that little is known of their effects on the host immune system.^299 302^ Second, despite considerable progress over the past two decades, our understanding of RCD-associated DAMP signaling remains limited. In particular, limited attention has been dedicated to the study of immunosuppressive DAMPs, including (but not limited to) phosphatidylserine, prostaglandin E_2_, and adenosine, especially in the context of ICD.^68 325–327^ Intriguingly, MAMPs can also be immunosuppressive, and these are important for the establishment of symbiosis.^328^ The impact of metabolism,^329^ the gut microbiota^330^ and the central nervous system^331^ on the release and activity of DAMPs also remains to be elucidated. Finally, the key role of the host in ICD detection has been mostly studied from an adjuvanticity perspective, that is, linked to the capacity of the host to decode DAMP signaling via PRRs.^5^ Conversely, little is known about the TCR repertoire of hosts that respond to ICD with robust adaptive immunity versus hosts that do not, and about the impact of environmental and behavioral features (eg, microbiome, dietary habits, stress) on host ICD sensing.

Along with the establishment of humanized rodent models that enable the investigation of ICD in vivo (although with the caveats of a murine microenvironment), we believe that these issues currently stand as the major challenges for the field in the near future. We surmise that the clinical efficacy of numerous agents currently employed for the management of cancer could be remarkably boosted if we acquire the capacity to use them as ICD inducers. Novel technologies are constantly improving our ability to monitor the immunological changes occurring in patients responding to ICD inducers, including alterations in the intratumoral and circulating TCR repertoire.^332^ Overall, the time is mature to take on the challenge to realize the clinical potential of ICD inducers and improve disease outcome for a variety of patients with cancer.
