# Correction: Consensus guidelines for the definition, detection and interpretation of immunogenic cell death

**DOI:** 10.1136/jitc-2019-000337corr1

**Published:** 2020-05-24

Galluzzi L, Vitale I, Warren S, *et al*. Consensus guidelines for the definition, detection and interpretation of immunogenic cell death. *J Immunother Cancer* 2020;8:e000337. doi: 10.1136/jitc-2019-000337

Since the online publication of this article, the authors have noticed that the author name Aitziber Buqué Martinez is abbreviated incorrectly. The author’s first name is Aitziber Buqué and the family name is Martinez. The correct abbreviation is Buqué Martinez A.

